# Comparative genomics of *Myxococcus* and *Pyxidicoccus*, including the description of four novel species: *Myxococcus guangdongensis* sp. nov., *Myxococcus qinghaiensis* sp. nov., *Myxococcus dinghuensis* sp. nov., and *Pyxidicoccus xibeiensis* sp. nov.

**DOI:** 10.3389/fmicb.2022.995049

**Published:** 2022-11-10

**Authors:** Chunling Wang, Yingying Lv, Lian Zhou, Yulian Zhang, Qing Yao, Honghui Zhu

**Affiliations:** ^1^Guangdong Academy of Sciences, Institute of Microbiology, Key Laboratory of Agricultural Microbiomics and Precision Application (MARA), Provincial Key Laboratory of Microbial Culture Collection and Application, Key Laboratory of Agricultural Microbiome (MARA), State Key Laboratory of Applied Microbiology Southern China, Guangzhou, China; ^2^College of Life Science, Huizhou University, Huizhou, China; ^3^College of Horticulture, South China Agricultural University, Guangdong Province Key Laboratory of Microbial Signals and Disease Control, Guangzhou, China

**Keywords:** *Myxococcus*, *Pyxidicoccus*, comparative genomics, novel species, CAZymes, BGCs

## Abstract

Myxobacteria are recognized for fascinating social behaviors and producing diverse extracellular active substances. Isolating novel myxobacteria is of great interest in the exploitation of new antibiotics and extracellular enzymes. Herein, four novel strains were isolated from Dinghu Mountain Biosphere Reserve, Guangdong province, and Qinghai virgin forest soils, Qinghai province, China. The phylogenetic analysis based on 16S rRNA gene and genomic sequences indicated that the four strains belong to the genera *Myxococcus* and *Pyxidicoccus*, sharing the highly similarities of 16S rRNA gene with the genera *Myxococcus* and *Pyxidicoccus* (99.3–99.6%, respectively). The four strains had average nucleotide identity (ANI) values of 82.8–94.5%, digital DNA–DNA hybridization (dDDH) values of 22.2–56.6%, average amino acid identity (AAI) values of 75.8–79.1% and percentage of conserved protein (POCP) values of 66.4–74.9% to members of the genera *Myxococcus* and *Pyxidicoccus*. Based on phylogenetic analyses, physiological and biochemical characteristics, and comparative genomic analyses, we propose four novel species of the genera *Myxococcus* and *Pyxidicoccus* and further supported the two genera above represented the same genus. Description of the four novel species is *Myxococcus guangdongensis* sp. nov. (K38C18041901^T^ = GDMCC 1.2320^T^ = JCM 39260^T^), *Myxococcus qinghaiensis* sp. nov. (QH3KD-4-1^T^ = GDMCC 1.2316^T^ = JCM 39262^T^), *Myxococcus dinghuensis* sp. nov. (K15C18031901^T^ = GDMCC 1.2319^T^ = JCM 39259^T^), and *Pyxidicoccus xibeiensis* sp. nov. (QH1ED-7-1^T^ = GDMCC 1.2315^T^ = JCM 39261^T^), respectively. Furthermore, comparative genomics of all 15 species of the genera *Myxococcus* and *Pyxidicoccus* revealed extensive genetic diversity. Core genomes enriched more genes associated with housekeeping functional classes while accessory genomes enriched more genes related to environmental interactions, indicating the former is relatively indispensable compared to signaling pathway genes. The 15 species of *Myxococcus* and *Pyxidicoccus* also exhibited great gene diversity of carbohydrate-active enzymes (CAZymes) and secondary metabolite biosynthesis gene clusters (BGCs), especially related to glycosyl transferases (GT2 and GT4), glycoside hydrolases (GH13 and GH23), non-ribosomal peptide synthetases (NRPS), and Type I polyketide synthase (PKS)/NRPS hybrids.

## Introduction

Myxobacteria are rod-shaped gram-negative bacteria and are distributed across the world (Mohr, 2018). Compared to research into their sophisticated social lifestyle, unstable morphological, and slow-growth characteristics (Shimkets et al., 2006; Awal et al., 2016), myxobacterial taxonomic research publications are relatively lacking. According to the latest taxonomic proposal, myxobacteria have been reclassified as the phylum *Myxococcota*, comprising the four orders *Myxococcales*, *Polyangiales*, *Nannocystales*, and *Haliangiales*, seven families, and 19 genera (Waite et al., 2020). The three genera *Myxococcus*, *Pyxidicoccus*, and *Corallococcus* of the family *Myxococcaceae* have often been isolated from various habitats, and their 16S rRNA genes show highly homologous (Mohr et al., 2016, 2017; Livingstone et al., 2017; Wang et al., 2020). Compared with the 98.65% of 16S rRNA gene sequence similarity, the 98.7% of *gyrB* gene sequence similarity is suitable as the threshold for species boundaries of the three genera above (Liu et al., 2021). With the advance of next-generation sequencing technologies, comparative genomics analysis has been applied to identify and classify myxobacterial species. Just recently, eight novel *Corallococcus*, three novel *Myxococcus*, and two novel *Pyxidicoccus* species were identified based on polyphasic taxonomy combined with pan-genomic analyses (Chambers et al., 2020; Livingstone et al., 2020).

Myxobacteria exhibit genome sizes ranging from 9.0 to 16.0 Mbp and the genomic DNA G + C contents around 70.0 mol% (Sharma et al., 2016; Sharma and Subramanian, 2017). The core genomes may meet myxobacterial requirements for gliding motility, group predation, multicellular fruiting body, and myxospore formation (Wrotniak-Drzewiecka et al., 2016). Moreover, myxobacteria show a wide range of predatory activity profiles against various bacteria and some fungi, but the action mode remains largely unknown (Thiery and Kaimer, 2020). Myxobacterial predatory activity seemed to be not congruent with phylogeny (Livingstone et al., 2017). Gliding motility, direct contact with prey, and lysis are required for predation (Thiery and Kaimer, 2020). Meanwhile, the outer membrane vesicles (OMVs) secreted by myxobacteria are packed with a cargo of secondary metabolites and hydrolytic enzymes. OMVs and OMV-free culture supernatant proved to kill prey cells (Evans et al., 2012; Berleman et al., 2014; Livingstone et al., 2018). Recently, a newly discovered type IV filament-like machinery (kil) that could promote myxobacterial motility arrest and prey cell plasmolysis has been proposed to be related to contact-dependent killing (Seef et al., 2021).

Herein, according to phylogenetic analyses, physiology and biochemical characterization, and genomic average nucleic acid identity (ANI), digital DNA–DNA hybridization (dDDH), average amino acid identity (AAI), and percentage of conserved protein (POCP) values, we propose four novel species of the *Myxococcus* and *Pyxidicoccus* and further support these two genera represented the same genus, which was proposed by Chambers et al. (2020). Furthermore, we carry out pan-genomics and compare the functional distribution of core, accessory, and unique genes of *Myxococcus* and *Pyxidicoccus.* We also analyze the diversity of carbohydrate-active enzymes (CAZymes) genes and secondary metabolite biosynthesis gene clusters (BGCs) of *Myxococcus* and *Pyxidicoccus*. This study provides four novel myxobacterial species for the exploitation of new secondary metabolites and extracellular enzymes and will deepen insights into the genetic and gene diversity of *Myxococcus* and *Pyxidicoccus*.

## Materials and methods

### Bacterial isolation and identification

Strains K15C18031901^T^ and K38C18041901^T^ were isolated from Dinghu Mountain Biosphere Reserve collected on 12 September 2017, located at Zhaoqing, Guangdong province, China, and strains QH3KD-4-1^T^ and QH1ED-7-1^T^ were isolated from Qinghai virgin forest soils collected on 6 October 2020, located at Xining, Qinghai province, China. The isolation method for myxobacteria was described by Wang C. L. et al. (2019) using *Escherichia coli* as baits. Isolates were picked on modified VY/2 agar (Baker’s yeast 5 g L^–1^, CaCl_2_⋅2H_2_O 1 g L^–1^, agar 15 g L^–1^, and pH 7.2), and a pure culture was obtained by the repeated subculture of cells from the edge of the colony. Upon purification, strains were stored at −80°C as suspensions containing 25% glycerol.

### Phylogenetic analysis based on 16S rRNA, *gyrB* genes, and genomic sequences

The genomic DNAs of four isolates above were extracted using the HiPure Bacterial DNA kit (Magen Biotech Co., Ltd., Guangzhou, China) according to the manufacturer’s instructions. The 16S rRNA gene was amplified using the genomic DNAs as templates and sequenced using the universal primers 27F and 1492R (Kai et al., 2006). The sequences obtained were submitted to similarity searches in the EzBioCloud^1^ and NCBI^2^ databases. The phylogenetic tree of 16S rRNA gene sequences was reconstructed based on the maximum-likelihood (ML) method with the model of Jukes–Cantor and ultrafast bootstrapping (-bb 1000) using the MEGA 7.0 software (Kumar et al., 2016).

The genomes were sequenced using the Illumina NovaSeq PE150 platform by Shanghai Majorbio Bio-Pharm Technology Co., Ltd. The clean data, generated from raw data by quality trimming, were assembled using the SPAdes (v3.11.1) with default parameters to obtain contigs and scaffolds. The genomic sequences of K15C18031901^T^, K38C18041901^T^, QH3KD-4-1^T^, and QH1ED-7-1^T^ were deposited at the NCBI^3^ database under the accession numbers JAKCFB000000000, JAJVKW000000000, JAKCFA000000000, and JAJVKV000000000, respectively. Furthermore, a phylogenomic tree was reconstructed based on 92 up-to-date core genes using the UBCG tools with a maximum-likelihood algorithm. In addition, *gyrB* genes were extracted from genome sequences, and sequence similarities were compared.

### Physiology characterization and fatty acid analyses

Fruiting bodies, swarming colonies, vegetative cells, and myxospores grown on VY/2 agar for 5–10 days at 30°C were observed and measured by stereomicroscope (Leica, M165C), field scanning electron microscope (SEM, HITACHI, S-3000N), and Nano Measurer 1.2 software, respectively. The gram staining reaction was performed by using the Gram Stain Kit according to the manufacturer’s instructions. The optimal temperatures for growth were investigated on VY/2 agar at 30, 35, 37, and 40°C for 5–7 days. Tolerance to NaCl concentrations (1–4%, at intervals of 1%, w/v, NaCl) and pH range (pH 5.0–9.0, at intervals of 1 unit) was performed on VY/2 agar at 30°C for 5–7 days. Enzymatic activities and substrate utilization were tested using the API ZYM and 20NE according to the manufactures’ instructions. Cellular fatty acids were extracted from cells grown on VY/2 agar at 30°C for 5 days using the Sherlock Microbial Identification System (MIDI) protocol (version 6.1). Fatty acids were analyzed by gas chromatography (model 7890A, Hewlett Packard) using the Sherlock Microbial Identification System (Sherlock version 6.1; TSBA6) (Sasser, 1990).

### Predation assays

The predatory activities of the four isolates were tested based on the method of Livingstone et al. (2017) using *Escherichia coli* ATCC 8739, *Micrococcus luteus* NCTC 2665, *Salmonella typhimurium* GDMCC 1.239, and *Staphylococcus aureus* GDMCC 1.1220 as prey. These prey were obtained from the Guangdong microbial culture collection center (GDMCC). The predatory zone on the prey lawn was observed after 3 days incubated at 30°C.

### Comparative genomic analyses

The other genomes of 11 type species within the genera *Myxococcus* and *Pyxidicoccus* were downloaded from the NCBI database for comparative analyses. The contigs (shorter than 500 bp) of used draft genomes above were removed. The completeness and contamination values of all 15 genomes were assessed by the software CheckM version 1.0.9. For consistency, the gene prediction and genomic annotation were reperformed using the software Prokka (v1.14.6) and the Rapid Annotation using Subsystem Technology (RAST) version 2.0^4^ with default parameters. The ANI and AAI among all species were calculated by fastANI (v1.32) and compareM (v0.1.2), respectively. The dDDH values were performed using Genome-to-Genome Distance Calculator (GGDC; v2.1).^5^ The POCP values were calculated as [(C1 + C2)/(T1 + T2)] × 100, where C represents the number of conserved proteins (identity, ≥40%; aligned length of query, ≥50%) and T represents the number of total proteins; 1 and 2 refer to input files 1 and 2, respectively (Qin et al., 2014). The ANI, dDDH, AAI, and POCP values were visualized using the “Heatmap” tool of the TBtools. The pan- and core genomes were calculated using OrthoFinder (v2.5.2) based on the “Diamond blastp with default parameters” and “MCL” algorithms to generate orthologous clusters. The representative sequences of each gene family were searched against the Kyoto Encyclopedia of Genes and Genomes (KEGG)^6^ database. In addition, the genes encoding CAZymes were identified using dbCAN2 meta server with HMMER annotation (E-Value < 1e-15, coverage > 0.35), and the BGCs were annotated using antiSMASH bacterial version 6.1.1 with default parameters, respectively. Identified clusters were further determined using MultiGeneBlast as previously described (Medema et al., 2013; Adamek et al., 2017).

## Results

### Phylogenetic analyses based on 16S rRNA, *gyrB* gene, and genomic sequences

**FIGURE 1:**
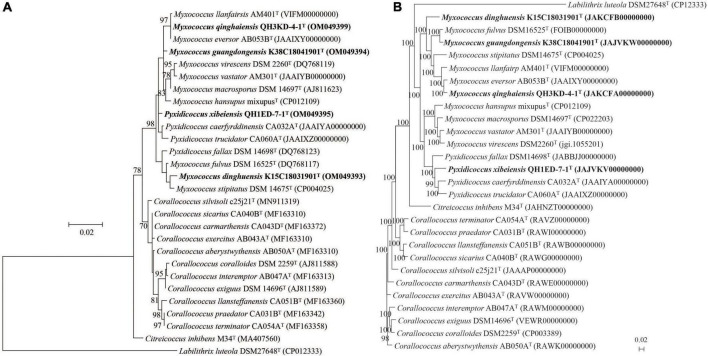
Phylogenetic trees among four putative species and their closely related species within the genera *Myxococcus*, *Pyxidicoccus*, and *Corallococcus*. **(A)** The ML tree based on 16S rRNA gene sequences is generated using the Jukes–Cantor model by the MEGA 7.0 software. **(B)** The UBCG phylogenomic tree based on 92 up-to-date bacterial core genes sequences is reconstructed using the ML algorithm. GenBank accession numbers are shown in parentheses. Bootstrap values (represented percentages of 1,000 replications) >70% are shown at nodes. *Labilithrix luteola* DSM 27648^T^ is used as an outgroup. Bar, 0.02 substitutions per nucleotide position.

The 16S rRNA gene sequences of strains K38C18041901^T^ (1398 bp; GenBank accession number: OM049394), K15C18031901^T^ (1432 bp; GenBank accession number: OM049393), QH3KD-4-1^T^ (1395 bp; GenBank accession number: OM049399), and QH1ED-7-1^T^ (1432 bp; GenBank accession number: OM049395) were obtained according to the methods described in the “Materials and methods” section. Strains K38C18041901^T^, K15C18031901^T^, and QH3KD-4-1^T^ showed the highest 16S rRNA gene sequence similarities of 99.4, 99.3, and 99.6%, respectively, with *Myxococcus fulvus* DSM 16525^T^, *Myxococcus stipitatus* DSM 14675^T^, and *Myxococcus eversor* AB053B^T^. Strain QH1ED-7-1^T^ exhibited the highest 16S rRNA gene similarity of 99.3% with *Pyxidicoccus trucidator* CA060A^T^ (Supplementary Table 1). Moreover, the ML phylogenetic tree reconstructed based on 16S rRNA gene sequences also indicated that the four strains above were affiliated with the genera *Myxococcus* and *Pyxidicoccus* (Figure 1A).

The genome lengths of four strains above were 10.5–12.8 Mbp with the genomic DNA G + C contents of 68.8–70.7 mol%. Other general genomic features of the four strains are listed in Supplementary Tables 1, 2. The genomic annotation result of the four strains by RAST pipeline is shown in Supplementary Figure 1. Furthermore, the phylogenomic tree suggested that strains K15C18031901^T^, K38C18041901^T^, and QH3KD-4-1^T^ formed a large clade with the genus *Myxococcus* and strain QH1ED-7-1^T^ formed a clade with the genus *Pyxidicoccus*. In addition, strain QH3KD-4-1^T^ was grouped with *M. eversor* AB053B^T^ and strain K38C18041901^T^ was grouped with *M. fulvus* DSM 16525^T^, respectively (Figure 1B). More specifically, four strains of the genus *Pyxidicoccus* formed a clade with four species of the genus *Myxococcus* (Figure 1B).

The *gyrB* gene sequences were extracted from genomic sequences. Strains K38C18041901^T^, K15C18031901^T^, QH3KD-4-1^T^, and QH1ED-7-1^T^ showed the highest *gyrB* gene sequence similarities of 94.0, 95.9, 91.4, and 93.7% with *M. fulvus* DSM 16525^T^, *M. stipitatus* DSM 14675^T^, *M. eversor* AB053B^T^, and *P. trucidator* CA060A^T^, respectively (Supplementary Table 1), all of which were lower than 98.7% threshold for species boundaries of three genera *Myxococcus*, *Pyxidicoccus*, and *Corallococcus* (Liu et al., 2021), indicating the four strains were potential new species.

### Genomic average nucleotide identity, digital DNA–DNA hybridization, amino acid identity, and percentage of conserved protein analyses

**FIGURE 2:**
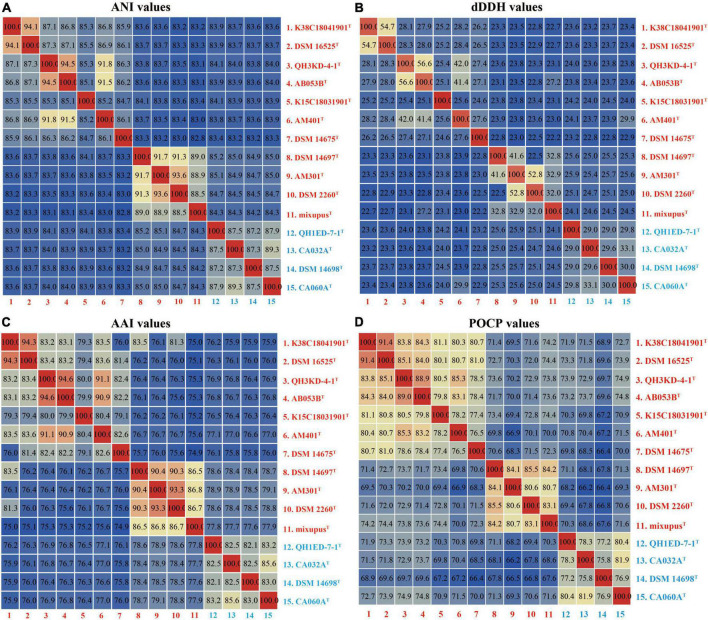
Heatmaps of the ANI **(A)**, dDDH **(B)**, AAI **(C)**, and POCP **(D)** values. The results are visualized using the “Heatmap” tool of the TBtools.

The ANI, dDDH, AAI, and POCP values were calculated to identify the genomic similarities of the four strains above with other species of the two genera *Myxococcus* and *Pyxidicoccus*. The four strains showed 82.8–94.5% ANI values with their closely related species, which were lower than the threshold value of 95–96% for species discrimination (Figure 2A; Richter and Rosselló-Móra, 2009). Moreover, the four strains exhibited 22.2–56.6% dDDH values with other related species, which were lower than the 70% cutoff value for species boundaries (Figure 2B; Chun et al., 2018). These results indicated the four strains above represent four novel genospecies. In addition, four strains of the genus *Pyxidicoccus* showed 75.8–79.1% AAI values with ten species of the genus *Myxococcus* (Figure 2C), which were higher than the threshold value of 65–72% for the genus boundaries (Konstantinos and James, 2007). Similarly, four strains of the genus *Pyxidicoccus* exhibited 66.4–74.9% POCP values with other species of *Myxococcus*, which were higher than 50% for the genus boundary (Qin et al., 2014). These results indicated members of the genus *Pyxidicoccus* were affiliated with the genus *Myxococcus*, as determined in the study by Chambers et al. (2020). Based on 16S rRNA and *gyrB* gene sequence similarities, genomic phylogenetic analyses, ANI, dDDH, AAI, and POCP values, the four strains represented four novel genospecies, and the two genera *Pyxidicoccus* and *Myxococcus* should belong to the same genus. Considering the close phylogenetic relationships, the type species of *M. fulvus* DSM 16525^T^, *M. eversor* AB053B^T^, *M. stipitatus* DSM 4675^T^, and *Pyxidicoccus fallax* DSM 14698^T^ were selected for physiochemical analyses.

### Physiology characterization and fatty acid analyses

The morphological characteristics of the four putative species grown on VY/2 agar are shown in Supplementary Figure 2. Fruiting bodies were series or single, roundish or oval (diameters of 150–470 μm), and multiple brightly colored grown on VY/2 agar (Supplementary Figures 2A–D). By gliding movement, colonies develop as thin, spreading swarms (Supplementary Figures 2E–H). All vegetative cells were gram-negative slender rod-shaped, measuring an average size of 0.30–0.45 μm × 2.5–6.0 μm, and formed circular myxospores (0.9–1.1 μm diameters) under field scanning electron microscope (Supplementary Figures 2I–M). Growth at different temperatures indicated that strains K38C18041901^T^, QH3KD-4-1^T^, and K15C18031901^T^ grew at 30°C, while their closely related species *M. fulvus* DSM 16525^T^, *M. eversor* AB053B^T^, and *M. stipitatus* DSM 14675^T^ grew at 35–40°C (Table 1). The optimum pH was 7.0 for all species with only three strains *M. fulvus* DSM 16525^T^, *M. eversor* AB053B^T^, and *M. stipitatus* DSM 14675^T^ exhibiting growth at pH 5.0 (Table 1). All strains can tolerate 1% (w/v) NaCl, while strains K38C18041901^T^ and K15C18031901^T^ can tolerate up to 2% (w/v) NaCl. Exceptionally, type species *M. eversor* AB053B^T^ can tolerate up to 4% (w/v) NaCl (Table 1). The biochemical results using API ZYM and 20NE tests are shown in Table 1. Strain QH1ED-7-1^T^ showed a similar activity profile with *P. fallax* DSM 14698^T^ except for acid phosphatase, β-glucosidase, and β-galactosidase. Strain K38C18041901^T^ was negative for *N*-acetyl*-*β*-*glucosaminidase but positive for β-galactosidase, while its closely related species *M. fulvus* DSM 16525^T^ was opposite. Strain QH3KD-4-1^T^ was negative for the utilization of β-glucosidase, arginine dihydrolase, and urease, while its closely related species *M. eversor* AB053B^T^ was all positive. Strain K15C18031901^T^ was negative for utilization of urease, while its closely related species were positive. Additional differences between the four strains and their related species are shown in Table 1. In addition, the four strains showed broad predatory abilities against prey *E. coli*, *M. luteus*, *S. typhimurium*, and *S. aureus* (Supplementary Figure 3).

**TABLE 1:** Physiology characteristics of the four strains and their closely related species.

Characteristics	1	2	3	4	5	6	7	8
35°C	–	+	–	+	–	+	+	+
37°C	–	+	–	+	–	–	+	+
40°C	–	–	–	+	–	–	W	+
pH 5.0	–	+	–	+	–	+	–	–
pH 9.0	–	–	–	+	–	+	+	+
2% NaCl	+	–	–	+	+	–	–	–
3% NaCl	–	–	–	+	–	–	–	–
4% NaCl	–	–	–	+	–	–	–	–
**API ZYM**								
Acid phosphatase	+	+	+	+	+	+	+	–
β-Glucosidase	+	+	–	+	+	–	+	–
*N*-acetyl-β-Glucosaminidase	–	+	–	–	–	+	–	–
**API 20NE**								
Nitrate reduction	+	+	–	–	–	–	–	–
Arginine dihydrolase	–	–	–	+	–	–	–	–
Urease	–	–	–	+	–	+	–	–
Gelatin hydrolysis	+	+	+	+	+	+	+	w
β-Galactosidase	+	–	+	+	+	+	–	+
**The G + C content (mol%)**	69.8	70.0	68.8	68.9	70.7	69.2	70.7	70.5

Strains: 1. *M. guangdongensis* K38C18041901^T^; 2. *M. fulvus* DSM 16525^T^; 3. *M. qinghaiensis* QH3KD-4-1^T^; 4. *M. eversor* AB053B^T^; 5. *M. dinghuensis* K15C18031901^T^; 6. *M. stipitatus* DSM 14675^T^; 7. *P. xibeiensis* QH1ED-7-1^T^; 8. *P. fallax* DSM 14698^T^. All strains can grow at 30°C and tolerate 1% NaCl. All strains are positive for alkaline phosphatase, esterase (C4), esterase (C8), lipase (C14), leucine arylamidase, valine arylamidase, cystine arylamidase, trypsin, *a*-chymotrypsin, naphthol-AS-BI-phosphohydrolase, and β-glucosidase. All strains are negative for α-galactosidase, β-galactosidase, β-glucuronidase, α-glucosidase, α-mannosidase, β-fucosidase, indole production, and glucose fermentation. +, positive; -, negative; w, weakly positive.

The major fatty acids (>5.0%) identified in the four strains and the closely related species included straight-chain fatty acids (SCFAs) of C_16:0_ ω*5c* (5.0–22.6%) and branched-chain fatty acids (BCFAs) of iso-C_15:0_ (19.4–36.7%) and iso-C_17:0_ (6.6–12.2%) (Table 2). The abundance of C_16:0_ ω*5c* and iso-C_15:0_, major biomarker fatty acids for members of the *Myxococcus* and *Pyxidicoccus* (Garcia et al., 2011), indicated that the four strains belonged to the genus *Myxococcus* and *Pyxidicoccus*. Besides, strain K38C18041901^T^ contained a lower amount of C_16:0_, summed feature 3 (C_16:1_ ω7*c* and/or C_16:1_ ω6*c*), and a higher amount of iso-C_15:0_ and summed feature 4 (iso-C_17:1_ I and/or anteiso-C_17:1_ B) than *M. fulvus* DSM 16525^T^. Strain K15C18031901^T^ contained a higher amount of C_16:0_ and iso-C_17:0_ than *M. stipitatus* DSM 14675^T^. Strain QH1ED-7-1^T^ contained a lower amount of C_16:0_ ω*5c*, C_16:0_ and a higher amount of summed features 3 and 5 (ante-C_18:0_ and/or C_18:2_ ω6*c*, ω9*c*) (Table 2). These results indicated that the four strains should belong to four novel species of the genera *Myxococcus* and *Pyxidicoccus*.

**TABLE 2:** Cellular fatty acids of the four strains and their closely related species.

Fatty acids	1	2	3	4	5	6	7
**Straight chain**							
C_9:0_	1.8	–	1.2	–	–	2.4	–
C_10:0_	–	1.2	1.8	1.0	1.2	–	1.4
C_14:0_	–	1.3	1.2	3.1	3.7	1.1	3.0
**C_16:1_ ω 5c**	**5.0**	**7.2**	**8.2**	**16.6**	**16.2**	**12.1**	**22.6**
C_16:0_	1.8	**6.7**	3.2	**12.0**	**5.4**	3.3	**8.1**
C_17:0_	–	–	–	–	–	3.0	–
C_16:0_ 3-OH	–	1.7	2.2	1.3	1.0	1.3	1.7
C_18:1_ ω9c	–	2.2	1.5	–	1.2	–	1.7
**Branched chain**							
iso-C_13:0_	3.2	1.3	–	1.1	–	–	–
**iso-C_15:0_**	**36.7**	**23.7**	**27.4**	**31.2**	**34.7**	**19.4**	**25.4**
iso-C_16:0_ H	1.0	–	–	–	–	4.1	**6.8**
iso-C_16:0_	2.3	2.3	2.1	1.6	1.0	1.0	1.2
anteiso-C_16:0_	1.6	–	–	2.0	–	–	–
iso-C_15:0_ 3-OH	3.8	2.7	3.2	1.2	2.6	2.3	2.4
iso-C_17:1_ ω5c	2.0	1.6	–	1.2	–	1.4	–
**iso-C_17:0_**	**7.5**	**8.6**	**7.7**	**12.2**	**7.7**	**8.0**	**6.6**
iso-C_18:0_	3.6	2.0	–	–	–	–	–
iso-C_17:0_ 3-OH	4.2	2.8	**5.1**	1.6	3.4	4.6	3.3
Summed feature 3	**9.3**	**20.5**	**13.0**	4.9	4.7	**14.8**	**7.6**
Summed feature 4	**6.7**	1.7	2.6	2.6	4.1	–	2.4
Summed feature 5	–	4.5	1.8	–	1.3	**9.3**	2.1

Strains: 1. *M. guangdongensis* K38C18041901^T^; 2. *M. fulvus* DSM 16525^T^; 3. *M. qinghaiensis* QH3KD-4-1^T^; 4. *M. dinghuensis* K15C18031901^T^; 5. *M. stipitatus* DSM 14675^T^; 6. *P. xibeiensis* QH1ED-7-1^T^; 7. *P. fallax* DSM 14698^T^. Major fatty acids (>10.0%) are indicated in bold; -, not detected or <1.0%. *Summed features are groups of two or three fatty acids that are not separated by GLC with the Sherlock Microbial Identification System. Summed feature 3 contained C_16:1_ ω7c and/or C_16:1_ ω6c. Summed feature 4 contained iso-C_17:1_ I and/or anteiso-C_17:1_ B. Summed feature 5 contained anteiso-C_18:0_ and/or C_18:2_ ω6c and/or C_18:2_ ω9c.

### Proposal of four novel species of the genera *Myxococcus* and *Pyxidicoccus*

Based on 16S rRNA and *gyrB* gene sequence similarities, genomic phylogenetic analyses, genomic ANI, dDDH, AAI, and POCP values, physiology characterizations, and fatty acid profiles, we propose four novel species: *Myxococcus guangdongensis* sp. nov. (type strain K38C18041901^T^, the same below), *Myxococcus dinghuensis* sp. nov. (K15C18031901^T^), *Myxococcus qinghaiensis* sp. nov. (QH3KD-4-1^T^), and *Pyxidicoccus xibeiensis* sp. nov. (QH1ED-7-1^T^), respectively. These analyses further supported that the two genera *Myxococcus* and *Pyxidicoccus* should be the same genus.

### Pan-genome analyses of the genera *Myxococcus* and *Pyxidicoccus*

**FIGURE 3:**
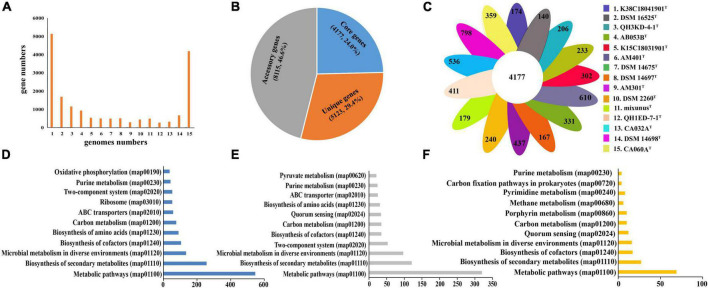
Distribution of gene families in the 15 genomes of the genera *Myxococcus* and *Pyxidicoccus*. **(A)** Numbers of gene families represented within the respective numbers of 15 genomes. **(B)** Numbers and percentages of core, accessory, and unique genes in 15 genomes. **(C)** Numbers of unique genes in each species. **(D–F)** Numbers of core genes **(D)**, accessory genes **(E)**, and unique genes **(F)** that associate with each indicated KEGG pathway maps.

General genomic characteristics of all 15 species of the genera *Myxococcus* and *Pyxidicoccus* are listed in Supplementary Table 2. Three of them were completely assembled with circle maps, whereas the others were draft genomes with multiple contigs and nucleic acid gaps. Furthermore, all of them were determined to be near-complete genomes with high completeness (>98%) and low contamination (<5.0%). The genome sizes ranged from 9.0 to 13.5 Mbp containing 7,258–10,697 genes with genomic DNA G + C contents of 68.7–70.7 mol%. By using “diamond blastp with default parameters” and “MCL” clustering, 132,667 putative protein-coding genes from 15 genomes were clustered into 17,415 gene families forming the pan-genome. We categorized the gene families based on the number of strains in each cluster. Core genes were present in all 15 genomes, accessory genes were present in 2–14 genomes, and unique genes were present only in a genome (Figure 3A). The pan-genome consisted of 4,177 (24.0%) core gene families, 8,115 (46.6%) accessory gene families, and 5,123 (29.3%) unique gene families (Figure 3B). Furthermore, the numbers of strain-specific genes ranged from 140 to 798, respectively (Figure 3C).

To compare the functional distribution of the core, accessory, and unique genes, we extracted protein sequences from the three sections to map to the KEGG Orthology (KO) database. In total, 48.9% (2012) of core entries, 20.0% (1621) of accessory entries, and only 3.9% (200) of unique entries were annotated. Most of the KO-annotated genes in the three genomes were related to metabolic pathways, especially biosynthesis of secondary metabolites, microbial metabolism in diverse environments, and biosynthesis of cofactors and amino acids (Figures 3E–G). In detail, the core genomes enriched more genes associated with housekeeping functional classes, such as ribosome and oxidative phosphorylation, while the accessory and unique genomes enriched more genes related to environmental interactions, such as quorum sensing (QS) (Figures 3D–F).

### The analyses of carbohydrate-active enzymes and biosynthesis gene clusters of the genera *Myxococcus* and *Pyxidicoccus*

**FIGURE 4:**
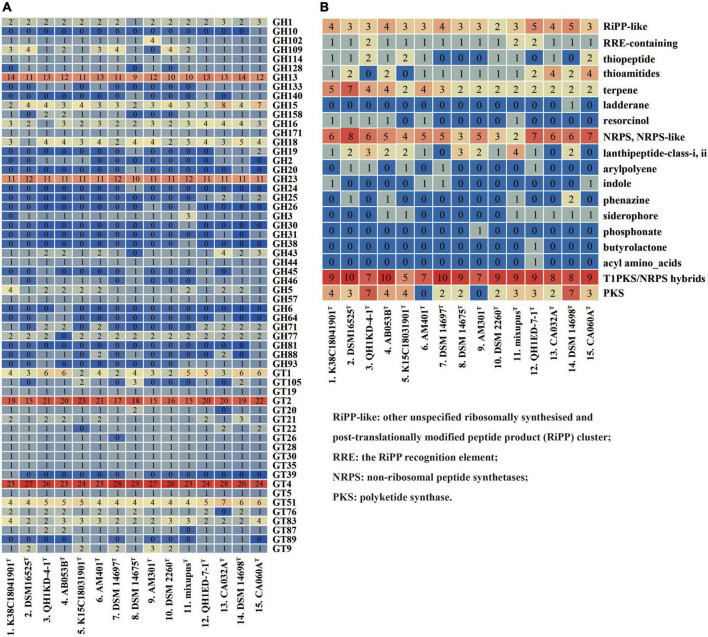
Type counts of GTs and GHs families **(A)** and BGCs **(B)** identified within the genera *Myxococcus* and *Pyxidicoccus*. RiPP-like, other unspecified ribosomally synthesized and post-translationally modified peptide product (RiPP) cluster; RRE, the RiPP recognition element; NRPS, non-ribosomal peptide synthetases; PKS, polyketide synthase.

We compared and screened genes encoding CAZymes in the 15 genomes. The pan-genome of the 15 species encoded 315 different CAZyme members, including 106 (33.7%) falling into core gene families, 184 (65.4%) falling into accessory gene families, and 25 (7.9%) falling into unique gene families (Supplementary Figure 4A). The types and numbers of glycosyl transferases (GTs) in core genes were much more than those in accessory genes (Supplementary Figures 4B,C). Further comparative analysis showed the 15 species encoded large numbers of GH13, GH23, GT2, and GT4 members (Figure 4A). These families were found to be putatively assigned to multiple enzyme activities, such as *a*-1,4-glucan branching enzyme (GH13), lysozyme type G, peptidoglycan lyase and chitinase (GH23), chitin and cellulose synthase (GT2), and sucrose synthase and *a*-glucosyltransferase (GT4). The results also exhibited multiple enzyme activities of the 15 species with predicted glucoamylase (GH18), glucodextranase (GH15), acetylgalactosaminidase (GH109), and galactosidase and glucosidase (GH16), though at a much lower abundance (Figure 4A).

Furthermore, antiSMASH server was used to predict BGCs of the 15 species. The results showed that all of the 15 species contained multiple gene clusters encoding other unspecified ribosomally synthesized and post-translationally modified peptide product (RiPP-like), terpene, non-ribosomal peptide synthetase (NRPS), NRPS-like, and Type I polyketide synthase (T1PKS)/NRPS hybrid types. Except for *Myxococcus llanfairp* AM401^T^ and *Myxococcus vastator* AM301^T^, the remaining 13 genomes contained different PKS gene clusters. Other multiple types, for example, arylpolyene, indole, phenazine, and butyrolactone, were contained in the unique or accessory genomes (Figure 4B). In addition, only four “most similar known clusters,” carotenoid (100% similarity), VEPE/AEPE/TG-1 (100% similarity), myxoprincomide-506, and alkylpyrone-407/393, were found in all 15 genomes, which were consistent with those described by Chambers et al. (2020). Moreover, except for *M. stipitatus* DSM 14675^T^ and *Myxococcus hansupus* mixupus^T^, the remaining 13 genomes contained gene cluster encoding myxochelin A/B. Moreover, the gene cluster encoding geosmin (100% similarity) was found in genomes of 14 species except *Pyxidicoccus xibeiensis* QH1ED-7-1^T^. The gene clusters and their similarities related to myxoprincomide-506, alkylpyrone-407/393, myxochelin A/B, geosmin, and carotenoid in different genomes are exhibited in Supplementary Figure 5. The results exhibited abundant gene diversity of CAZymes and BGCs in the 15 species of *Myxococcus* and *Pyxidicoccus*.

## Discussion

This study isolated and identified four new species within the genera *Myxococcus* and *Pyxidicoccus* and supported the two genera should be the same genus (Figures 1, 2). Furthermore, pan-genomics indicated that all 15 species of *Myxococcus* and *Pyxidicoccus* clustered into 17,415 orthologous groups, which were divided into 24.0% core gene families, 46.6% accessory gene families, and 29.4% unique gene families (Figures 3A–C) using software OrthoFinder with default parameters. However, because of multiple contigs of some used draft genomes, big genes can be split across contigs and the real gene numbers might be overestimated for the pan-genomics. In addition, according to the described study (Chambers et al., 2020), the pan-genome of 11 species of *Myxococcus* and *Pyxidicoccus* contained 64,700 orthologous clusters analyzed by software Roary, which were higher than our data, maybe because of the use of lower cutoffs by the software Roary. Core genomes enriched more genes related to housekeeping functional classes such as ribosome and oxidative phosphorylation, while the accessory and unique genomes enriched more genes associated with environmental interactions, such as quorum sensing (Figures 3D–F). A reasonable interpretation is that housekeeping genes are relatively indispensable compared to signaling pathway genes, and therefore, signaling genes will be enriched in the accessory genome. Moreover, bacterial QS is a mechanism by which bacteria exchange intracellular or intercellular information, coordinate population behavior, and regulate gene expression (Xiao et al., 2022). Myxobacteria appear to shape genetic diversity by communicating with intracellular or intercellular information to broaden accessory and unique genomes.

It is known that myxobacteria exhibit a broad predatory ability to various bacteria, but the predation efficiencies are different (Livingstone et al., 2017). Lytic enzymes and secondary metabolites secreted by myxobacteria play important roles in predatory process (Xiao et al., 2011; Arend et al., 2020). Based on our results, the four novel species also showed the broad predatory ability against prey (Supplementary Figure 3). In addition, the abundant genes encoding CAZymes were found to mainly lie in accessory genome of the 15 species (Supplementary Figure 4). The GTs and GHs are the two richest CAZymes families, mainly included large numbers of GT2, GT4, GH13, and GH23 members (Figure 4A), exhibiting a variety of enzyme activities. Furthermore, multiple BGCs types: RiPP-like, terpene, NRPS, NRPS-like, and T1PKS/NRPS hybrids, were distributed in all 15 genomes, while only four “Most similar known clusters” were common, including alkylpyrone-407/393, carotenoid, myxoprincomide-506, and VEPE/AEPE/TG-1 (Figure 4B). Alkylpyrone-407/393 is type III PKS, revealing potent topoisomerase activity *in vitro* (Hug et al., 2019). Carotenoids are lipophilic isoprenoid pigments that protect cells from damage and death by quenching highly reactive and toxic oxidative species (Padmanabhan et al., 2021). Myxoprincomide-506 is a NRPS/PKS natural product, which has been shown to be involved in the predatory killing of some prey (Cortina et al., 2012; Berleman et al., 2014; Müller et al., 2016). Furthermore, myxochelin A/B and geosmin are found in 13 or 14 genomes (Figure 4B). Myxochelin A/B is a siderophore released under iron-deficient conditions to form a complex with iron (Lee et al., 2020). Geosmin has been found in various strains of cyanobacteria, streptomyces, and myxobacteria, which is considered a major cause of most taste and odor episodes in drinking water (Wang Z. J. et al., 2019). Recently, geosmin has been found to appear to act as a warning signal indicating the unpalatability of its producers and reducing predation in a manner that benefits predator and prey (Zaroubi et al., 2022). Different CAZymes and secondary metabolites produced by the genera *Myxococcus* and *Pyxidicoccus* may to a certain extent reflect their predation efficiencies against various bacteria and fungi.

## Description of *Myxococcus guangdongensis* sp. nov.

*Myxococcus guangdongensis* (guang.dong.en’sis. N. L. fem. adj. *guangdongensis*, referring to Guangdong, a province of southern China, where the type strain was isolated).

Vegetative cells are gram-negative bacilli tapering slightly at the ends, appearing 0.3–0.4 μm × 2.2–4.1 μm under scanning electron microscope. Colonies are swarming and pale yellow on VY/2 agar. Fruiting bodies are irregular spheroids, large scattered, and orange-yellow (diameters of 150–350 μm). Myxospores are rounded spherical (diameters of 0.9–1.0 μm). Aerobic growth at 30°C but not 35°C, at pH 6.0–8.0 but not pH 5.0 or 9.0, and with up to 2.0% (w/v) NaCl. Positive for nitrate reduction and β-glucosidase. The major fatty acids (>5.0%) are C_16:0_ ω5c, iso-C_15:0_, iso-C_17:0_, and summed features 3 and 4. Cells prey upon *E. coli*, *M. luteus*, *S. typhimurium*, and *S. aureus*.

The type strain, K38C18041901^T^ (=GDMCC 1.2320^T^ =JCM 39260^T^), was isolated from forest soils collected from Dinghu Mountain Biosphere Reserve, Guangdong Province, China (23°10′24′′N; 112°32′17′′E). Genomic G + C content is 69.8 mol%. The accession number of the genome is JAJVKW000000000.

## Description of *Myxococcus qinghaiensis* sp. nov.

*Myxococcus qinghaiensis* (qing.hai.en’sis. N.L. fem. adj. *qinghaiensis*, referring to Qinghai, a province of north-west China, where the soil sample containing the type strain was collected).

Vegetative cells are gram-negative bacilli tapering slightly at the ends, appearing 0.3–0.4 μm × 2.5–4.5 μm. Colonies are swarming and pale yellow on VY/2 agar. Fruiting bodies are irregular spheroids, stacked in series, and pale yellow (diameters of 220–470 μm). Myxospores are rounded spherical (diameters of 0.9–1.0 μm). Aerobic growth at 30°C but not 35°C, at pH 6.0–8.0 but not pH 5.0 or 9.0, and with up to 1.0% (w/v) NaCl. Negative for β-glucosidase. The major fatty acids (>5.0%) are C_16:0_ ω5c, iso-C_15:0_, iso-C_17:0_, iso-C_17:0_ 3-OH, and summed feature 3. Cells prey upon *E. coli*, *M. luteus*, *S. typhimurium*, and *S. aureus*.

The type strain, QH3KD-4-1^T^ (=GDMCC 1.2316^T^ =JCM 39262^T^), was isolated from forest soils collected from Qinghai Virgin Forest, Qinghai Province, China (37°0′52′′N; 102°12′5′′E). The genomic G + C content is 68.8 mol%. The accession number of the genome is JAKCFA000000000.

## Description of *Myxococcus dinghuensis* sp. nov.

*Myxococcus dinghuensis* (ding.hu.en’sis. N.L. fem. adj. *dinghuensis*, referring to Dinghu Mountain, Guangdong Province, China, where the soil sample containing the type strain was collected).

Vegetative cells are gram-negative bacilli tapering slightly at the ends, appearing 0.3–0.4 μm × 2.1–4.5 μm. Colonies are swarming and pale silver on VY/2 agar. Fruiting bodies are irregular spheroids, deep-yellow (diameters of 180–370 μm). Myxospores are rounded spherical (diameters of 0.9–1.1 μm). Aerobic growth at 30°C but not 35°C, at pH 6.0–8.0 but not pH 5.0 or 9.0, and with up to 2.0% (w/v) NaCl. Positive for β-glucosidase. The major fatty acids (>5.0%) are C_16:0_ ω5*c*, C_16:0_, iso-C_15:0_, and iso-C_17:0_. Cells prey upon *E. coli*, *M. luteus*, *S. typhimurium*, and *S. aureus*.

The type strain, K15C18031901^T^ (=GDMCC 1.2319^T^ =JCM 39259^T^), was isolated from forest soils collected from Dinghu Mountain Biosphere Reserve, Guangdong Province, China (23°10′24′′N; 112°32′10′′E). The genomic DNA G + C content is 70.7 mol%. The accession number of the genome is JAKCFB000000000.

## Description of *Pyxidicoccus xibeiensis* sp. nov.

*Pyxidicoccus xibeiensis* (xi.bei.en’sis. N.L. masc. adj. *xibeiensis*, referring to Xibei, the Chinese phoneticization for north-west China, where the soil sample was collected).

Vegetative cells are gram-negative bacilli tapering slightly at the ends, appearing 0.3–0.5 μm × 5.0–6.6 μm. Colonies are swarming and bright metal color on VY/2 agar. Fruiting bodies are irregular spheroids and metal luster (diameters of 150–390 μm). Myxospores are rounded spherical (diameters of 0.9–1.0 μm). Aerobic growth up to 40*^o^*C, at pH 6.0–9.0 but not pH 5.0, and with up to 1.0% (w/v) NaCl. Positive for β-glucosidase and negative for β-Galactosidase. The major fatty acids (>5.0%) are C_16:0_ ω5c, iso-C_15:0_, iso-C_17:0_, and summed features 3 and 5. Cells prey upon *E. coli*, *M. luteus*, and *S. typhimurium*, but not *S. aureus*.

The type strain, QH1ED-7-1^T^ (=GDMCC 1.2315^T^ = JCM 39261^T^), was isolated from forest soils collected from Qinghai Virgin Forest, Qinghai Province, China (37°0′52′′N; 102°12′5′′E). The genomic DNA G + C content is 70.7 mol%. The accession number of the genome is JAJVKV000000000.

## Data availability statement

The datasets presented in this study can be found in online repositories. The names of the repository/repositories and accession number(s) can be found in the article/Supplementary material.

## Author contributions

CW: conceptualization, project administration, data curation, software, visualization, and writing—original draft. YL: formal analysis, methodology, and project administration. LZ and YZ: methodology and project administration. QY and HZ: writing—review and editing. All authors contributed to the article and approved the submitted version.

## Footnotes

1
http://eztaxon-e.ezbiocloud.net/

2
www.ncbi.nlm.nih.gov

3
https://www.ncbi.nlm.nih.gov/genome

4
http://rast.nmpdr.org/

5
http://ggdc.dsmz.de/

6
https://www.kegg.jp/
