# Supplementary material for: Comparative genomics of Myxococcus and Pyxidicoccus, including the description of four novel species: Myxococcus guangdongensis sp. nov., Myxococcus qinghaiensis sp. nov., Myxococcus dinghuensis sp. nov., and Pyxidicoccus xibeiensis sp. nov
Source: Front Microbiol. 2022 Nov 10;13:995049. doi: 10.3389/fmicb.2022.995049 (PMC9684338; doi:10.3389/fmicb.2022.995049)

Supplementary tables and figures

Comparative genomics of *Myxococcus* and *Pyxidicoccus*, including the description of four novel species: *Myxococcus guangdongensis* sp. nov., *Myxococcus qinghaiensis* sp. nov., *Myxococcus dinghuensis* sp. nov. and *Pyxidicoccus xibeiensis* sp. nov.

Chunling Wang[1, 2], Yingying Lv[1], Lian Zhou[1], Yulian Zhang[1], Qing Yao[3] and Honghui Zhu[1*]

[1]Key Laboratory of Agricultural Microbiomics and Precision Application (MARA), Guangdong Provincial Key Laboratory of Microbial Culture Collection and Application, Key Laboratory of Agricultural Microbiome (MARA), State Key Laboratory of Applied Microbiology Southern China, Institute of Microbiology, Guangdong Academy of Sciences, Guangzhou 510070, Guangdong, P. R. China

[2]College of life science, Huizhou University, Huizhou 516007, Guangdong, P. R. China

[3]College of Horticulture, South China Agricultural University, Guangdong Province Key Laboratory of Microbial Signals and Disease Control, Guangzhou 510642, Guangdong, P. R. China

Running title: Four novel *Myxococcus* species

Corresponding author: Honghui Zhu; +86-020-87685669; zhuhh_gdim@163.com

TABLE S1 General features of the four strains.

Strains	Samples	Sources	Locations	Closest type strain (16S rRNA)	16S rRNA gene similarity (%)	gyrB gene similarity (%)	16S rRNA accession numbers
K38C18041901	Dinghu mountain forest soil	Zhaoqing, China	23º10'24" N; 112º32'17" E	*Myxococcus fulvus* DSM 16525^T	99.4	94.0	OM049394
K15C18031901	Dinghu mountain forest soil	Zhaoqing, China	23º10'24" N; 112º32'10" E	*Myxococcus stipitatus* AB053B^T	99.3	95.9	OM049393
QH3KD-4-1	Qinghai virgin forest soil	Qinghai, China	37º0'52"N; 102º12'5"E	*Myxococcus eversor* AB053B^T	99.6	91.4	OM049399
QH1ED-7-1	Qinghai virgin forest soil	Qinghai, China	37º0'52"N; 102º12'5"E	*Pyxidicoccus trucidator* CA060A^T	99.3	93.7	OM049395

TABLE S2 General characteristics of 15 genomes within the genera *Myxococcus* and *Pyxidicoccus*.

Species	Accession numbers	Taxonomic affiliation	Size (Mbp)	%GC	Gene numbers	Contigs	N50 (bp)	Completeness (%)	Contamination (%)	Strain heterogeneity (%)
Myxococcus eversor AB053B^T	JAAIXY00000000	*Myxococcus*	11.4	68.9	9124	96	313,084	99.4	2	33.3
Myxococcus fulvus DSM 16525^T	FOIB00000000	*Myxococcus*	10.8	70.0	8581	42	755,538	99.4	0.7	0
Myxococcus llanfairp AM401^T	VIFM00000000	*Myxococcus*	12.4	68.7	10032	677	35,723	99.4	4.3	0
Myxococcus macrosporus DSM 14697^T	CP022203	*Myxococcus*	9.0	70.6	7258	1	-	98.7	1.3	0
Myxococcus stipitatus DSM 14675^T	CP004025	*Myxococcus*	10.4	69.2	8087	1	-	99.4	0	0
Myxococcus vastator AM301^T	JAAIYB00000000	*Myxococcus*	9.0	69.9	7455	946	15,871	98.1	2.6	0
Myxococcus virescens DSM 2260^T	jgi.1055201.1	*Myxococcus*	9.2	69.1	7528	57	503,429	99.4	1.3	0
Myxococcus hansupus mixupus^T	CP012109	*Myxococcus*	9.5	69.2	7644	1	-	98.7	0.7	0
Myxococcus guangdongensis K38C18041901^T	JAJVKW000000000	*Myxococcus*	11.2	69.8	8921	53	554,740	99.4	0.7	0
Myxococcus dinghuensis K15C18031901^T	JAKCFB000000000	*Myxococcus*	10.5	70.7	8439	124	207,354	99.4	3.4	0
Myxococcus qinghaiensis QH3KD-4-1^T	JAKCFA000000000	*Myxococcus*	11.7	68.8	9178	88	323,385	99.4	2	0
Pyxidicoccus xibeiensis QH1ED-7-1^T	JAJVKV000000000	*Pyxidicoccus*	12.8	70.7	9824	51	1,094,250	100	1.7	0
Pyxidicoccus caerfyrddinensis CA032A^T	JAAIYA00000000	*Pyxidicoccus*	13.4	70.2	10385	132	321,034	100	2.6	0
Pyxidicoccus fallax DSM 14698^T	JABBJJ00000000	*Pyxidicoccus*	13.5	70.5	10679	764	31,267	99.7	3.9	0
Pyxidicoccus trucidator CA060A^T	JAAIXZ00000000	*Pyxidicoccus*	12.7	70.3	9751	82	375,293	100	2.1	0

FIGURE S1 The genomes annotations of strains K38C18031901^T, K15C18041901^T, QH3KD-3-1^T and QH1ED-7-1^T by the RAST pipeline.

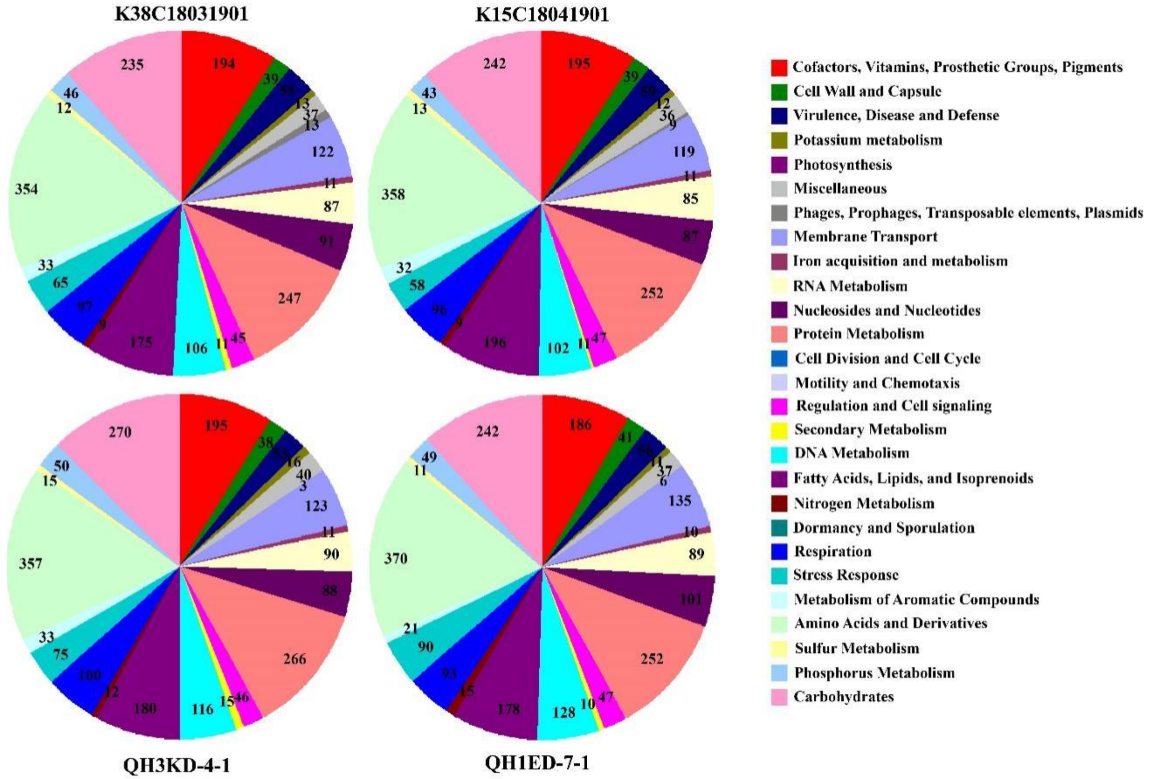

FIGURE S2 Morphologies of strains K15C18031901^T, K38C18041901^T, QH3KD-4-1^T and QH1ED-7-1^T. Pictures of fruiting bodies (A-D) and swarms (E-H). Bars = 250 µm (A, C) and 500 µm (B, D-H). Scanning electron micrographs of vegetative cells and myxospores (I-M). Bars = 2.5 µm. Photographs are taken from a 5-10 d culture.

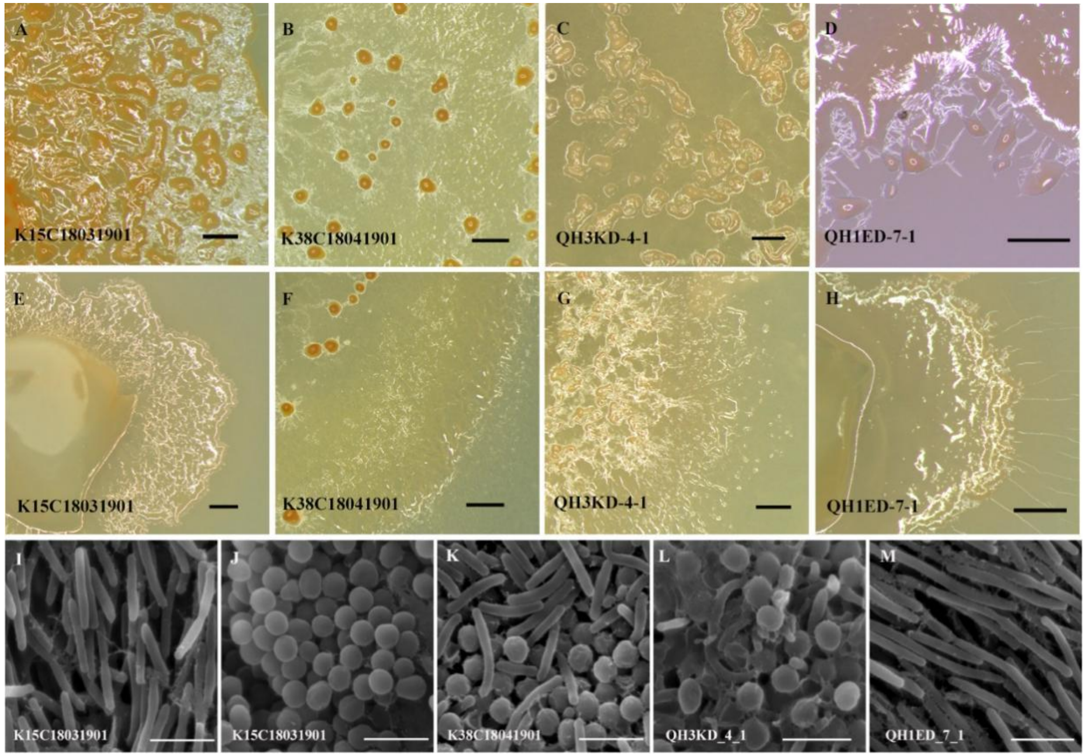

FIGURE S3 Predation behavior of the four candidate species against four prey: *Escherichia coli* ATCC 8739, *Micrococcus luteus* NCTC 2665, *Salmonella typhimurium* GDMCC 1.239 and *Staphylococcus aureus* GDMCC 1.1220. Photographs were obtained by stereomicroscope after 3 d incubated at 30°C. Bar = 2 mm.

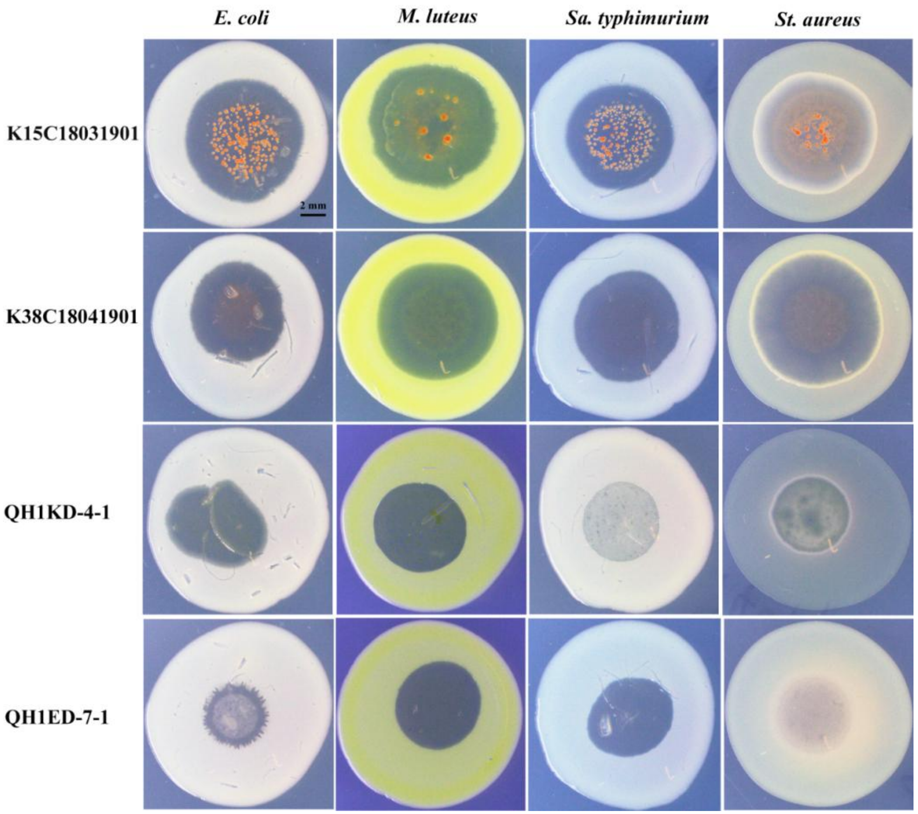

FIGURE S4 The distribution and types of CAZymes in different sections of the 15 genomes. (A). Numbers and percentages of CAZymes in core, accessory and unique genes. The Numbers (B) and Types (C) of six classes in core, accessory and unique genes.

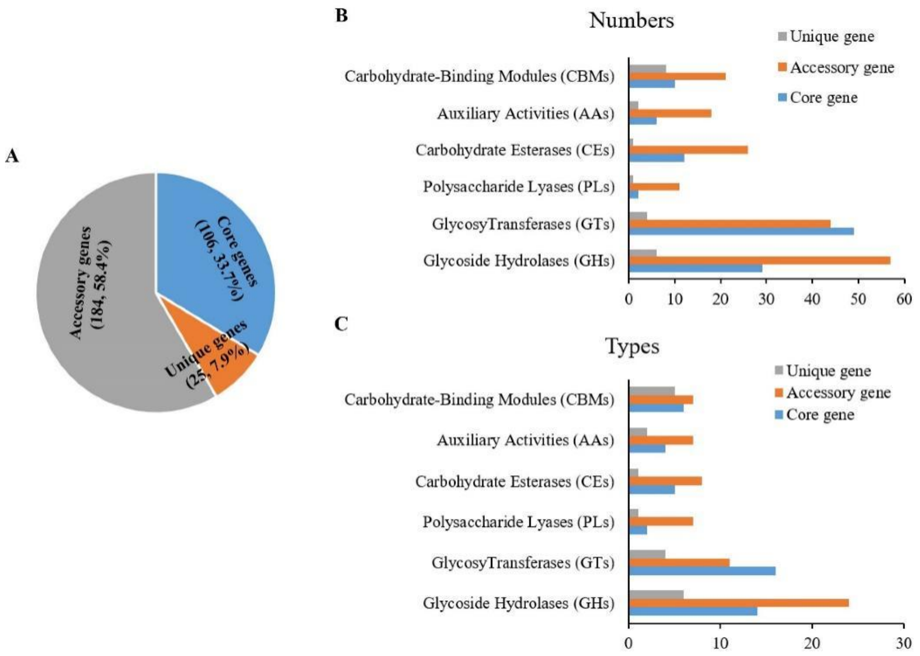

FIGURE S5 The gene clusters and their similarities related to myxoprincomide-506, alkylpyrone-407/393, myxochelin A/B, geosmin and carotenoid in different genomes within the genera *Myxococcus* and *Pyxidicoccus*.

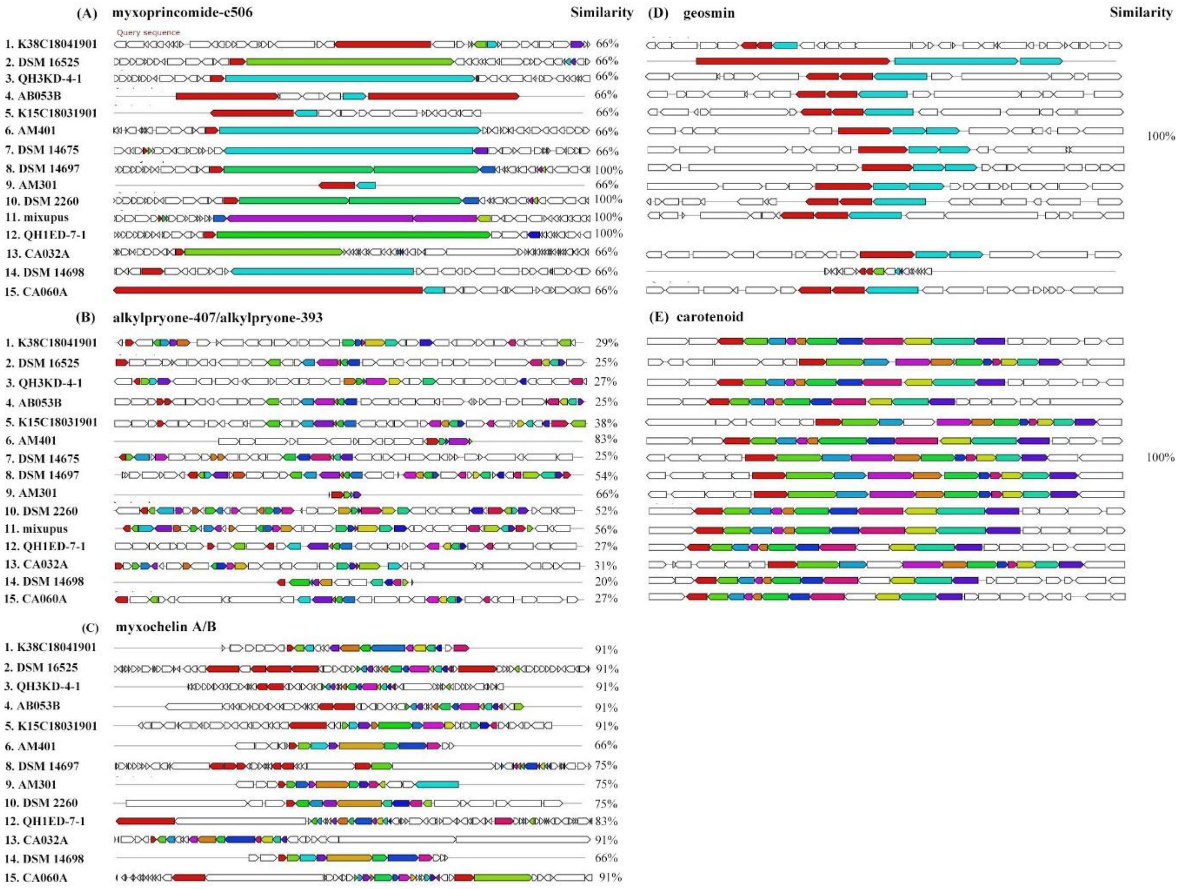

Supplement: Supplementary file 1 [file Data_Sheet_1.pdf]
